# Facilitating Digital Transformation in Nursing Through Nursing Development Units: Scoping Review

**DOI:** 10.2196/89051

**Published:** 2026-06-04

**Authors:** Annabell Gangnus, Pascal Müller, Katrin Gayen, Uta Kirchner-Heklau, Sebastian Hofstetter, Patrick Jahn

**Affiliations:** 1 Department of Internal Medicine, Health Service Research Working Group, Acute Care, Faculty of Medicine, University Medicine Halle (Saale), Martin-Luther-University Halle-Wittenberg, Magdeburger Straße 12, Halle (Saale), 06112, Germany, 49 3455576593; 2 Dorothea-Erxleben-Learning-Centre, Faculty of Medicine, University Medicine Halle (Saale), Martin-Luther-University Halle-Wittenberg, Halle (Saale), Germany

**Keywords:** nursing development unit, professional development, innovation in nursing, reflective practice, digital transformation, interdisciplinary collaboration, organizational learning, PRISMA

## Abstract

**Background:**

Nursing Development Units (NDUs) are structured clinical environments designed to enhance professional development, collaboration, and organizational learning. While NDUs have been widely studied for their impact on nursing practice, their role in supporting digital transformation in health care has been less explicitly examined.

**Objective:**

This scoping review aimed to map the characteristics, structures, and processes of NDUs and identify how these elements may relate to digital readiness and the integration of digital tools in nursing practice.

**Methods:**

We conducted a scoping review of 40 publications describing NDUs. Data were coded iteratively to identify patterns at the individual, interpersonal, and organizational levels. Core elements and subcategories of NDUs were mapped, highlighting potential connections to capacities required for digital transformation.

**Results:**

NDUs operate across three levels: (1) individual—fostering professional role development, leadership, and resilience; (2) interpersonal—promoting participatory cultures, collaborative practice, and shared unit visions; and (3) organizational—providing structured frameworks for practice philosophy, role development, resource and change management, and embedded evaluation systems. While most primary publications did not explicitly address digital competencies, the structures, reflective practices, and collaborative processes described represent foundational capacities for engaging with digital tools, innovation, and organizational change.

**Conclusions:**

NDUs are characterized by multilevel structures and processes that enhance professional and organizational capacities. Although explicit evidence on digital transformation is limited, these capacities align with key prerequisites for the adoption and effective use of digital technologies in nursing practice. Further research is needed to examine how NDUs directly support digital innovation in clinical settings.

## Introduction

Digital transformation is no longer a future scenario in health care but a present reality driven by artificial intelligence, remote monitoring, electronic health records, and data-informed decision-making [1-4]. While these innovations promise improved care quality, efficiency, and patient engagement, their implementation in nursing faces persistent challenges, including digital burnout, workflow disruption, resistance to change, and insufficient staff-centered design [5,6]. These barriers are not merely technical but rooted in organizational culture, leadership, and professional identity. Consequently, digital transformation in nursing often fails due to misalignment between technological innovations and clinical practice [6]. In this review, digital transformation in nursing is understood as a sociotechnical process. It involves technological adoption, changes in professional roles and competencies, and the adaptation of organizational routines and culture.

Nursing Development Units (NDUs) have emerged as a promising model to foster innovation and support digital transformation in nursing. NDUs are nursing-led, practice-based innovation hubs designed to integrate research, education, and clinical practice through reflective, participatory, and evidence-informed development cycles [7-9]. Unlike isolated pilot projects, NDUs are embedded within health care organizations and supported by dedicated leadership, resources, and evaluation systems, enabling long-term iterative learning and continuous improvement [10,11]. Their core mechanisms—structured reflection, participatory decision-making, and embedded evaluation—directly address known barriers to digital transformation by empowering nurses as cocreators of practice change.

Despite this potential, NDUs remain undertheorized in the context of digital health. Systematic mapping of their conceptual features, functional roles, and organizational frameworks—particularly regarding how they enable or constrain digital transformation—is lacking. Moreover, terminological variation (eg, “Clinical Development Unit,” “Practice Development Unit,” and “Nursing Professional Unit”) complicates cross-contextual comparisons and limits the transferability of insights [11-13]. This conceptual and methodological heterogeneity highlights a key research gap: the absence of a coherent, internationally applicable framework to understand how NDUs function as catalysts for digital innovation in nursing.

This scoping review aimed to address this gap by systematically mapping the international literature on NDUs, synthesizing their core conceptual features, functional roles, and organizational frameworks. The review also explored how NDUs engage with digital transformation—not as passive recipients of technology but as active spaces of knowledge translation [14], organizational change [15], and co-design [16]. A scoping review methodology was chosen due to the conceptual complexity, terminological diffusion, and limited existing synthesis of the NDU concept [17,18]. This approach allows for a comprehensive, transparent, and structured mapping of the literature, providing a foundation for future implementation models and strategies for digital innovation in nursing.

## Methods

This scoping review followed the Joanna Briggs Institute methodology [17] and the framework by Arksey and O’Malley [18], which encompasses 4 phases: identifying relevant publications, selecting publications, extracting data, and synthesizing findings. Reporting adhered to the PRISMA-ScR (Preferred Reporting Items for Systematic Reviews and Meta-Analyses extension for Scoping Reviews) checklist to ensure transparency and reproducibility [19].

### Search Strategy

A systematic literature search was conducted in May 2025 in the databases MEDLINE (via PubMed), ScienceDirect, EBSCO, Cochrane Library, and Europe PMC. Supplementary searches in Google Scholar and LIVIVO captured gray literature and publications not indexed in biomedical databases. Results were sorted by relevance, and all retrieved records were screened without a stopping rule to maximize sensitivity.

The primary database search used the exact phrase “Nursing Development Unit” to prioritize conceptual precision. No additional filters (eg, publication year, study design, or language) were applied to capture the complete literature on NDUs. This decision was grounded in the historically established use of the term. In the literature, “NDU” often denotes a defined organizational model rather than a generic practice development activity. We anticipated that broader search strings (eg, including “Practice Development Unit” or “Clinical Development Unit”) would markedly increase retrieval in the main databases. Much of this literature is conceptually adjacent but heterogeneous (eg, general practice development, quality improvement, or clinical education units) and does not necessarily reflect the NDU model targeted in this review.

To reduce the risk of missing relevant publications due to terminological variation, we complemented the core database search with supplementary searches and screened for records that provided structural or process descriptions consistent with NDUs (eg, “Clinical Development Unit,” “Practice Development Unit,” “Nursing Clinical Development Unit,” and “Nursing Professorial Unit”). The full MEDLINE search syntax is provided in Multimedia Appendix 1.

Publications were included if they described structural, functional, or organizational characteristics of NDUs, as well as development processes and implementation strategies. Publications primarily evaluating effectiveness or outcomes (eg, patient metrics and clinical performance) were excluded as the focus was on conceptual mapping rather than outcome assessment. Disease- or population-specific NDUs were included only if they provided insights into NDU structures or processes. Only English- or German-language publications with available full texts were considered. English was used to ensure coverage of the international literature, whereas German-language publications were included for feasibility given the review team’s proficiency. Textbox 1 summarizes the eligibility criteria.

Textbox 1. **Inclusion and exclusion criteria.**

**Inclusion criteria**

- Empirical and conceptual publications with abstracts available
- Language: German or English
- Description of content-related aspects, progressive development process, and implementation strategies of Nursing Development Units (NDUs)

**Exclusion criteria**

- Disease-specific focus (unless NDU structures or processes were described)
- Evaluation of NDUs (effectiveness or outcomes)
- Full text not available

### Publication Selection and Data Extraction

Duplicate records were manually removed based on title, author, digital object identifier, journal, and year. Three reviewers (AG, PM, and KG) independently screened titles and abstracts, and any record deemed eligible by at least one reviewer progressed to full-text review. The involvement of 3 reviewers at this stage was chosen to efficiently manage the large number of retrieved records.

Full-text screening was performed independently by 2 reviewers (AG and KG) using predefined inclusion and exclusion criteria, with disagreements resolved through discussion. All included publications were full-text documents (peer-reviewed articles or gray literature reports) that underwent thematic analysis. Data extraction followed a standardized charting framework capturing structural, functional, and organizational characteristics; development and implementation processes; and references to digital transformation where present. Each publication was extracted by a single reviewer, with the framework ensuring consistency. Given the more in-depth nature of full-text assessment, the involvement of 2 reviewers was considered sufficient to ensure consistency and methodological rigor.

**Figure 1.**
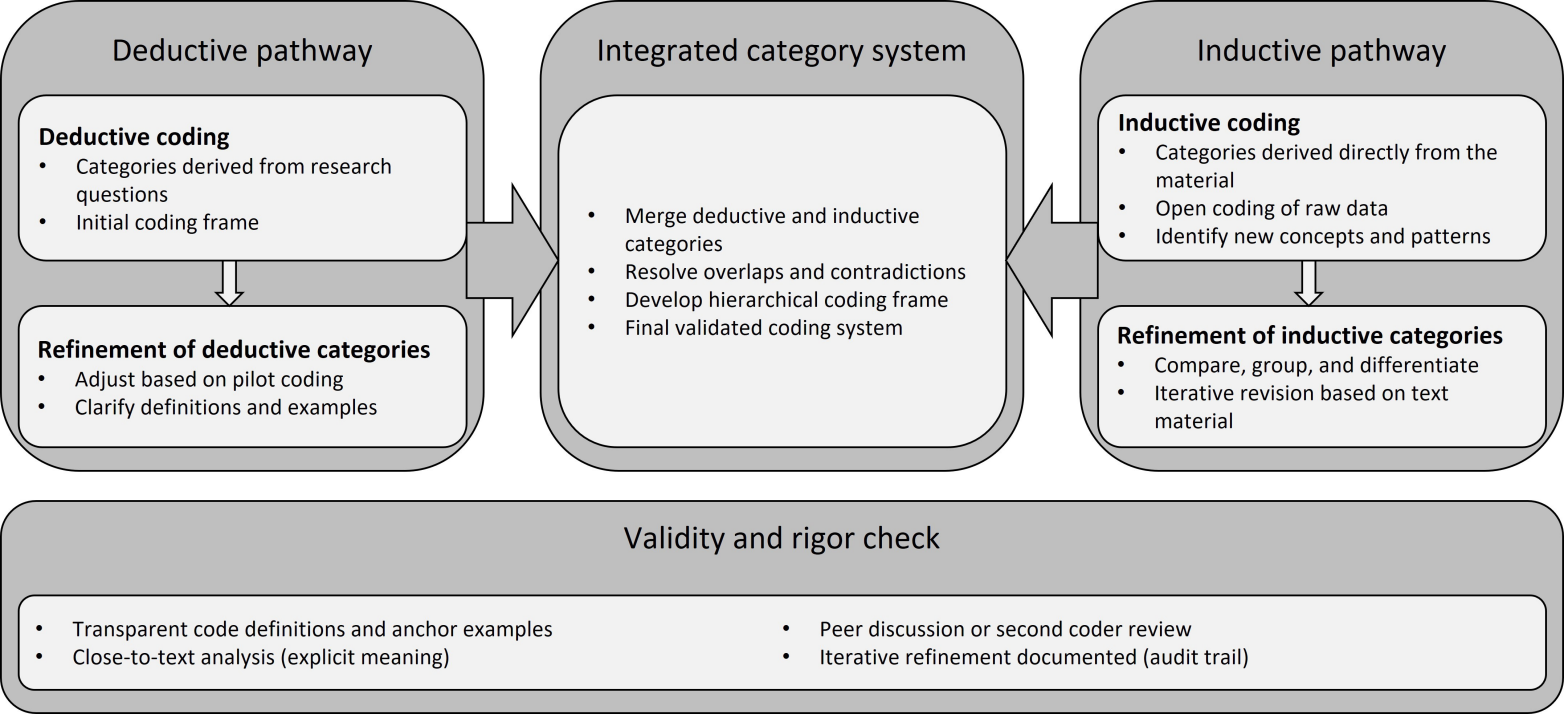
Qualitative content analysis process according to Kuckartz and Rädiker [20] (applied in this review).

A qualitative synthesis was conducted using the structured qualitative content analysis by Kuckartz and Rädiker [20], as recommended by Levac et al [21]. Thematic categories emerged through an iterative process: (1) deductive mapping of initial categories from the research questions (eg, “leadership identity”), (2) inductive coding of all publications to identify recurring patterns, and (3) aggregation of overlapping patterns into the final thematic structure through team consensus. Category definitions, anchor examples, and iterative coding refinement ensured analytical rigor. Figure 1 [20] illustrates the process.

### Ethical Considerations

As this study was a scoping review of published literature, it did not involve human participants, and therefore, ethics approval was not required.

## Results

### Publication Selection

**Figure 2.**
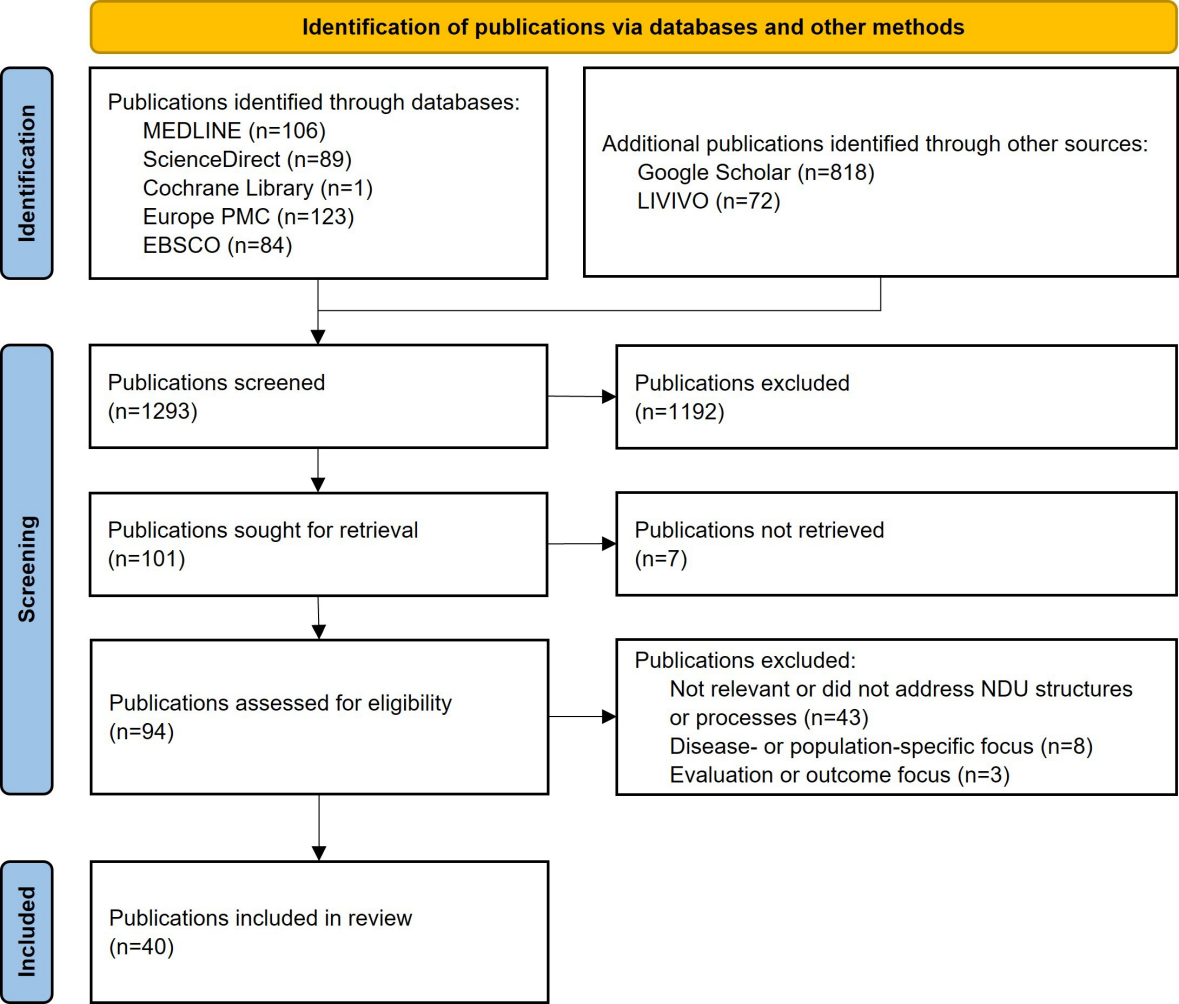
PRISMA (Preferred Reporting Items for Systematic Reviews and Meta-Analyses) flowchart illustrating the study selection process (adapted from Moher et al [22]). NDU: Nursing Development Unit.

The database search conducted in May 2025 yielded 1293 records: 106 (8.2%) from MEDLINE (via PubMed), 818 (63.3%) from Google Scholar, 72 (5.6%) from LIVIVO, 89 (6.9%) from ScienceDirect, 1 (0.1%) from the Cochrane Library, 123 (9.5%) from Europe PMC, and 84 (6.5%) from EBSCO. After removing duplicates and screening titles and abstracts, of the 1293 publications, 101 (7.8%) underwent full-text review. Applying the predefined eligibility criteria resulted in 40 publications included for in-depth analysis. No unresolved disagreements occurred during the selection process. The publication selection process is illustrated in the PRISMA (Preferred Reporting Items for Systematic Reviews and Meta-Analyses) flowchart (Figure 2 [22]).

### Main Characteristics of the Included Publications

The 40 included publications originated from the United Kingdom (n=26, 65%) and Australia (n=14, 35%) and covered the period from 1991 to 2017. Most publications were in English (n=39, 97.5%), with 2.5% (n=1) in German. Study designs comprised qualitative, quantitative, mixed methods, and conceptual approaches. The publications examined how professional roles, leadership, collaborative practices, organizational structures, and reflective processes shape nursing practice. Publication characteristics are summarized in Table 1.

**Table 1.** Characteristics of the included publications (N=40).

Study	Country	Design	Focus	Purpose
Atsalos and Greenwood [8], 2001	Australia	Qualitative	Leadership experiences in NDUs^a^	To explore leadership experiences of nurses working in NDUs under conditions of organizational stress
Atsalos et al [23], 2007	Australia	Qualitative	Experiences of nurse leaders in NDUs	To explore the experiences of nurse leaders involved in developing Clinical Development Units into centers of nursing excellence
Avallone and Gibbon [24], 1998	United Kingdom	Cross-sectional survey	Nurses’ perceptions of their work environment in an NDU	To examine nurses’ perceptions of their work environment within an NDU
Bell and Procter [25], 1998	United Kingdom	Qualitative	Experiences of nurses working in an NDU	To explore nurses’ experiences of involvement in nursing research within an NDU
Bland [10], 1997	United Kingdom	Case study	Development of an emergency nurse practitioner role	To describe the development of the emergency nurse practitioner role within an accident and emergency NDU
Bowles and Bowles [26], 2000	United Kingdom	Quantitative	Transformational leadership in NDUs vs conventional units	To compare transformational leadership in NDUs and conventional clinical settings
Cannard [27], 1996	United Kingdom	Quantitative	Aromatherapy to promote relaxation and stress reduction	To describe the implementation of aromatherapy as a practice development initiative within an NDU
Christensen and Craft [28], 2017	Australia	Conceptual	Translating research into nursing practice	To discuss strategies for translating nursing research into practice through an NDU
Christian and Norman [29], 1998	United Kingdom	Qualitative	Clinical leadership in NDUs	To examine approaches to clinical leadership development within NDUs in England
Draper [30], 1996	United Kingdom	Narrative review	Opportunities for evaluation of NDUs	To critically examine the purposes, characteristics, and effectiveness of NDUs
Duffield [31], 2005	Australia	Evaluation study	Masterclass for unit managers	To evaluate the design and delivery of a leadership masterclass for nursing unit managers
Gerrish [7], 2001	United Kingdom	Evaluation study	Evaluation of NDUs	To evaluate a Nursing and Practice Development Unit accreditation program
Gerrish and Ferguson [32], 2000	United Kingdom	Qualitative	Factors influencing NDU progress	To identify factors influencing the development and progress of NDUs
Graham [33], 1996	United Kingdom	Conceptual	Conceptual framework for NDUs	To present a conceptual framework supporting reflective practice and practice development within NDUs
Graham [34], 2000	United Kingdom	Qualitative	Reflective practice in mental health nurses	To examine reflective practice processes among mental health nurses working in an NDU
Graham [35], 2003	United Kingdom	Qualitative	Leadership perspectives in NDUs	To explore leadership perspectives within an NDU from academic and clinical viewpoints
Greenwood [36], 2000	Australia	Qualitative	Clinical Development Units: challenges and issues	To describe the characteristics, achievements, and challenges of Clinical Development Units
Greenwood [12], 1997	Australia	Qualitative	Accreditation of NDUs	To describe NDUs and examine models of accreditation
Greenwood [37], 1999	Australia	Case study	Western Sydney approach	To describe the establishment and leadership preparation approach of a Clinical Development Unit network in western Sydney, New South Wales
Greenwood and Kearns [38], 1996	Australia	Qualitative	Establishing a transcultural NDU	To describe the establishment of a transcultural NDU in an Australian context
Greenwood [13], 2000	Australia	Qualitative	Issues surrounding establishment and survival of NDUs	To examine challenges related to the establishment and sustainability of NDUs
Greenwood and Parsons [39], 2002	Australia	Evaluation study	Evaluation of a leadership preparation program	To evaluate a leadership preparation program for Clinical Development Unit leaders
Happell and Martin [40], 2002	Australia	Qualitative	Changing mental health nursing culture	To describe the implementation of a nursing Clinical Development Unit program in mental health nursing
Happell and Martin [41], 2004	Australia	Evaluation study	Evaluation of the Nursing Clinical Development Unit program	To evaluate outcomes of a Nursing Clinical Development Unit program in mental health nursing
Happell and Martin [42], 2005	Australia	Evaluation study	Changing the culture of mental health nursing	To assess the impact of a Nursing Clinical Development Unit program on mental health nursing culture
Johns [43], 1991	United Kingdom	Conceptual	Holistic model of nursing practice	To present a holistic model of nursing practice developed within an NDU
Keatinge and Scarfe [44], 1998	United Kingdom	Qualitative	NDUs in dementia care	To describe the establishment of an NDU in dementia care
Keatinge et al [45], 2000	United Kingdom	Action research	Nursing management of agitation in institutionalized residents with dementia	To examine nursing management of agitation in dementia care through participatory action research within an NDU
Malby [46], 1996	United Kingdom	Qualitative	Overview of NDUs in the United Kingdom	To provide an overview of the development and implementation of NDUs in the United Kingdom
Manley [47], 1997	United Kingdom	Action research	Advanced practitioner and consultant nurse role	To develop and examine an advanced practitioner role through action research within an NDU context
Parsons and Mott [11], 2003	Australia	Qualitative	Toward Clinical Development Units	To describe the principles and processes underpinning Clinical Development Units
Pearson [48], 1997	United Kingdom	Evaluation study	King’s Fund Centre NDU network	To assess the progress and direction of the King’s Fund Centre NDU network
Redfern and Stevens [49], 1998	United Kingdom	Qualitative	NDU structure and orientation	To describe the structure, aims, and organization of NDUs
Redfern and Murrells [50], 1998	United Kingdom	Qualitative	Research, audit, and networking activity in NDUs	To compare research, audit, and networking activities between NDUs and non-NDUs
Redfern et al [51], 1997	United Kingdom	Qualitative	Evaluation of NDUs	To evaluate the value and core characteristics of NDUs
Ryan [52], 1994	United Kingdom	Qualitative	Improving discharge planning	To describe practice development activities aimed at improving discharge planning within an NDU
Schiereck [53], 2000	United Kingdom	Qualitative	Social interaction in NDUs	To examine social interactions between nurses and patients within an NDU
Scholes [54], 1996	United Kingdom	Qualitative	Role transition and emotional labor	To explore the impact of working in an NDU on practitioners’ role transition and emotional labor
Vaughan [9], 1998	United Kingdom	Qualitative	History of NDU programs	To compare the development trajectories of 2 NDU programs
Wright [55], 2007	United Kingdom	Qualitative	Contribution to quality	To examine the concept of NDUs and their contribution to quality in nursing practice

a NDU: Nursing Development Unit.

### Core Elements of NDUs

#### Overview

**Figure 3.**
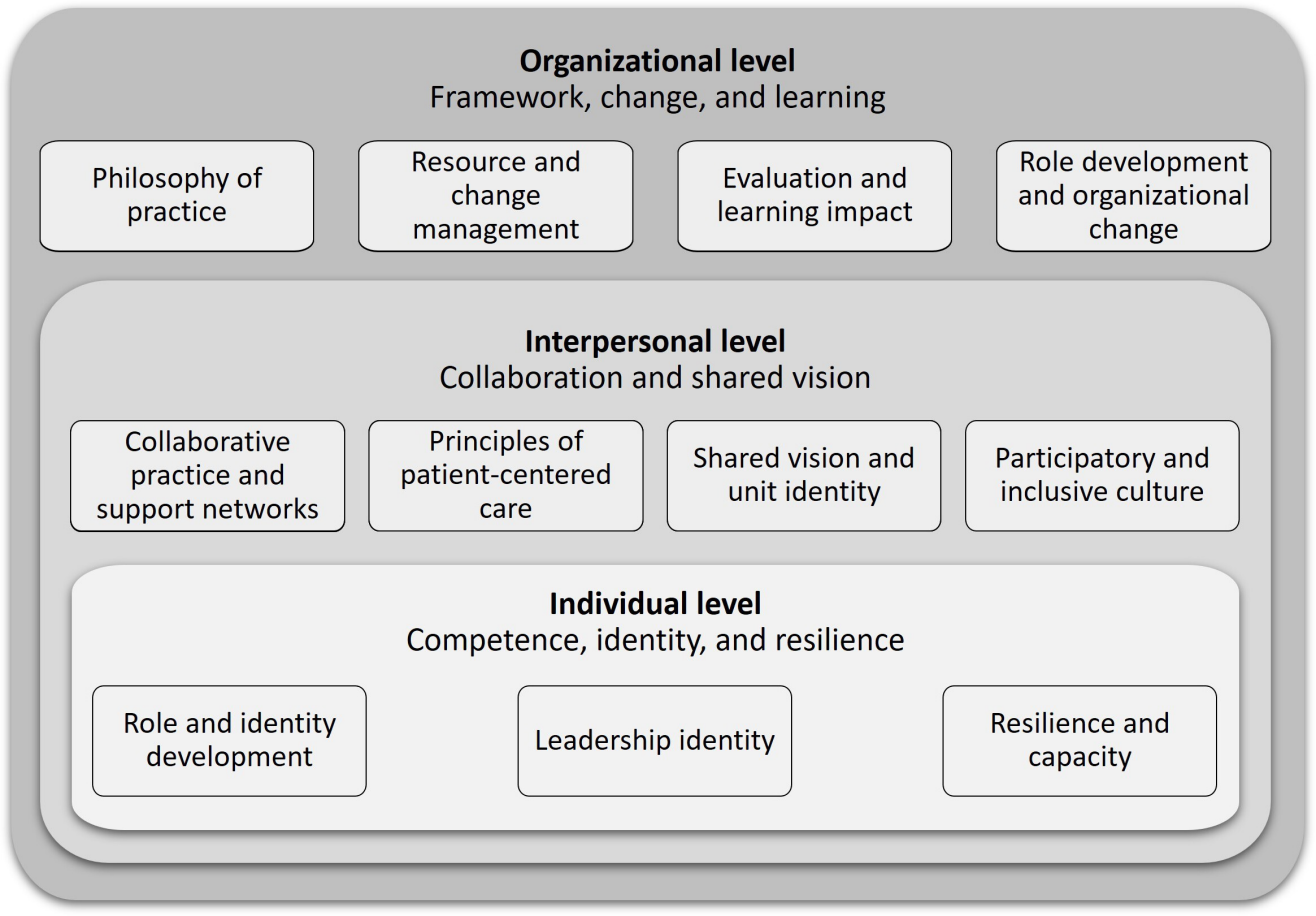
Core elements of a Nursing Development Unit inductively synthesized from the included publications.

The literature consistently described NDUs as operating across 3 interrelated levels: individual, interpersonal, and organizational (Figure 3). These levels structure descriptions of professional development, collaboration, and organizational learning. This framework emerged inductively from the data through iterative coding of all included publications, reflecting patterns observed in the literature rather than a pre-established theoretical model.

Several characteristics identified at these levels—such as competence development, participatory practices, and embedded learning structures—are discussed in the broader literature on organizational readiness for change and digital transformation. While explicit engagement with digital technologies was seldom reported in the included publications, the identified capacities and practices indicated potential enablers for adopting digital tools and supporting digital innovation in nursing.

#### Individual Level: Competence, Identity, and Resilience

NDUs were described as settings in which professional roles, leadership capabilities, and personal resilience are addressed. Activities included reflective practice, integration of ethical and evidence-based principles, and adaptation to evolving clinical demands. Table 2 maps publications contributing to individual-level components.

A clear understanding of professional roles, the integration of personal and shared philosophies of practice, and the development of self-efficacy and professional coherence were described as central elements of role and identity development [23,43]. Several publications highlighted intrinsic motivation and self-awareness in relation to personal growth and competence development [23].

**Table 2.** Mapping publications to individual-level components of Nursing Development Units (n=17).

Level and subcategory	Publications
Individual	
Role and identity development	[8,10,23,25,33-35,43,45,47,54]
Leadership identity	[8,23,26,29,31,35,39]
Resilience and capacity	[27,34,44,45,54]

Reflective practice was described as integrating ethical considerations, clinical judgment, and personal values into everyday care [43]. The literature reported structured forms of reflection, including written protocols, workshops, and reflective models, which were used to support critical inquiry into values, roles, and routines and articulate emerging professional identities within NDUs [33,34]. Modular training programs addressing clinical and research competencies were described across several publications and commonly linked to supervision and assessment processes [10].

Leadership identity was addressed in a subset of publications (7/17, 41.2%), which described clinical leaders as taking on facilitative and innovative roles and as supporting team processes. At the same time, challenges such as variable staff motivation and uncertainty regarding the NDU concept were reported [8,23]. Leadership support structures, including mentorship, networking, and formal development programs, were described in relation to team support during periods of change [31].

Several publications (5/17, 29.4%) described NDUs as contexts in which professional resilience is addressed, particularly in relation to iterative development processes and challenges associated with time constraints, emotional labor, and organizational obstacles [23,43]. Staff perceptions reported in the literature included high expectations, impatience for visible progress, and difficulties adjusting to evolving roles [43].

Across the included publications, reflective practice, continuous competence development, and leadership identity were recurrently described at the individual level. While the reviewed publications did not explicitly address digital competencies or digital practices, they outlined professional capacities—such as critical reflection, adaptability, and role clarity—that are frequently discussed in the broader literature in relation to organizational and technological change.

#### Interpersonal Level: Collaboration and Shared Vision

Across the included publications, NDUs were described as fostering participatory and inclusive cultures, supporting collaborative practices, and facilitating the development of shared visions and unit identities. Several publications reported that team interactions and decision-making processes emphasized principles of patient-centered care. Table 3 summarizes the publications contributing to the interpersonal-level subcategories.

Participatory and inclusive cultures were described as involving collaboration, shared decision-making, and ongoing improvement [32,53]. Publications reported that traditional nursing boundaries were sometimes transcended, enabling staff to engage in collective reflection, articulate unit philosophies, formulate action plans, and evaluate progress. Bottom-up approaches, including staff participation as coresearchers, were described in relation to collective ownership of practice change [32,45].

**Table 3.** Mapping publications to interpersonal-level components of Nursing Development Units (n=30).

Level and subcategory	Publications
Interpersonal	
Participatory and inclusive culture	[8,23-25,32,34,40,42,43,53,54]
Collaborative practice and support networks	[24,25,34,37,39,41,45,47,50,52,53]
Shared vision and unit identity	[9,11-13,28,33,36-38,40,42,44,46,49]
Principles of patient-centered care	[8,27,38,43,44,52,53]

Collaborative practices and support networks were described as developing through mentorship and interdisciplinary teamwork, supporting staff in addressing challenges and continuing professional growth [23,49]. Strategic planning processes were reported to clarify team needs, anticipate obstacles, and link practice activities to evidence generation. Several publications described collaborative planning as fostering shared ownership and a collective vision for change [54]. Partnerships with academic institutions were reported to support training, research, and innovation [9,49].

Shared vision and unit identity were described as contributing to team coherence. Staff involvement in defining the NDU’s purpose, intended outcomes, and guiding principles was frequently reported [43,45,54]. The literature highlighted that shared philosophies of practice grounded in clinical realities and staff perspectives can reinforce team coherence and collective identity [43]. Exposure to experiences from other NDUs was reported to help manage expectations, facilitate collective problem-solving, and support ongoing development [44].

Patient-centered care was described as emphasizing trust, sensitivity, shared decision-making, and patient autonomy [8,43]. Evidence-based approaches were reported across NDUs, although implementation varied. Some publications reported extensive evaluation frameworks, whereas others focused on routine questioning and evidence-informed decisions [9]. Integration with relational care models such as primary nursing was described as an example of applying patient-centered principles in practice [8,43,53].

Across the reviewed publications, participatory cultures, collaborative planning, and shared decision-making were consistently reported as central interpersonal characteristics of NDUs. Although digital technologies were not explicitly addressed, these interactional patterns reflected approaches commonly discussed in the broader literature for co-designing and implementing complex interventions, including digital tools in health care settings.

#### Organizational Level: Framework, Change, and Learning

At the organizational level, NDUs were described as involving frameworks and structures that support practice development, change, and learning. Several publications highlighted overarching philosophies of practice, structured role development, and embedded evaluation systems as recurring elements. Table 4 summarizes the publications contributing to the organizational-level subcategories.

**Table 4.** Mapping publications to organizational-level components of Nursing Development Units (n=31).

Level and subcategory	Publications
Organizational	
Philosophy of practice	[7,9,11-13,28,33,35-38,40-44,46,48-51,55]
Resource and change management	[9,12,13,26,31,32,36-39,41,44,46,48,49,51,55]
Role development and organizational change	[10,11,29,33,35,37,43,47,51]
Evaluation and learning impact	[7,28,30-32,39,41,48,50-53,55]

Philosophies of practice were described as providing frameworks of values, guiding principles, and standards that inform unit culture and practice [51]. Role development and organizational change were reported in relation to clarifying responsibilities, establishing decision-making processes, and supporting structural adjustments to avoid duplication and facilitate innovation [33,53].

Management of resources and change was described in the literature as including strategies to allocate time and materials, address resistance, and respond to operational pressures [49,53]. Evaluation and learning systems were reported to support reflective processes, facilitate improvements based on evidence, and connect practice to research and learning across disciplines [50,51,53]. Iterative development processes using structured strategies, participatory approaches, and strategic planning were described as approaches to respond to local needs while maintaining growth and improvement [10,11,29,50,51,53,54].

Embedded evaluation systems and iterative learning cycles were frequently reported as integral to NDU development. While these publications did not specifically address digital innovations, the described organizational infrastructures and learning mechanisms were consistent with approaches that are widely considered relevant for implementing and adapting complex interventions, including digital tools in health care settings.

### Development and Implementation Process of NDUs

**Figure 4.**
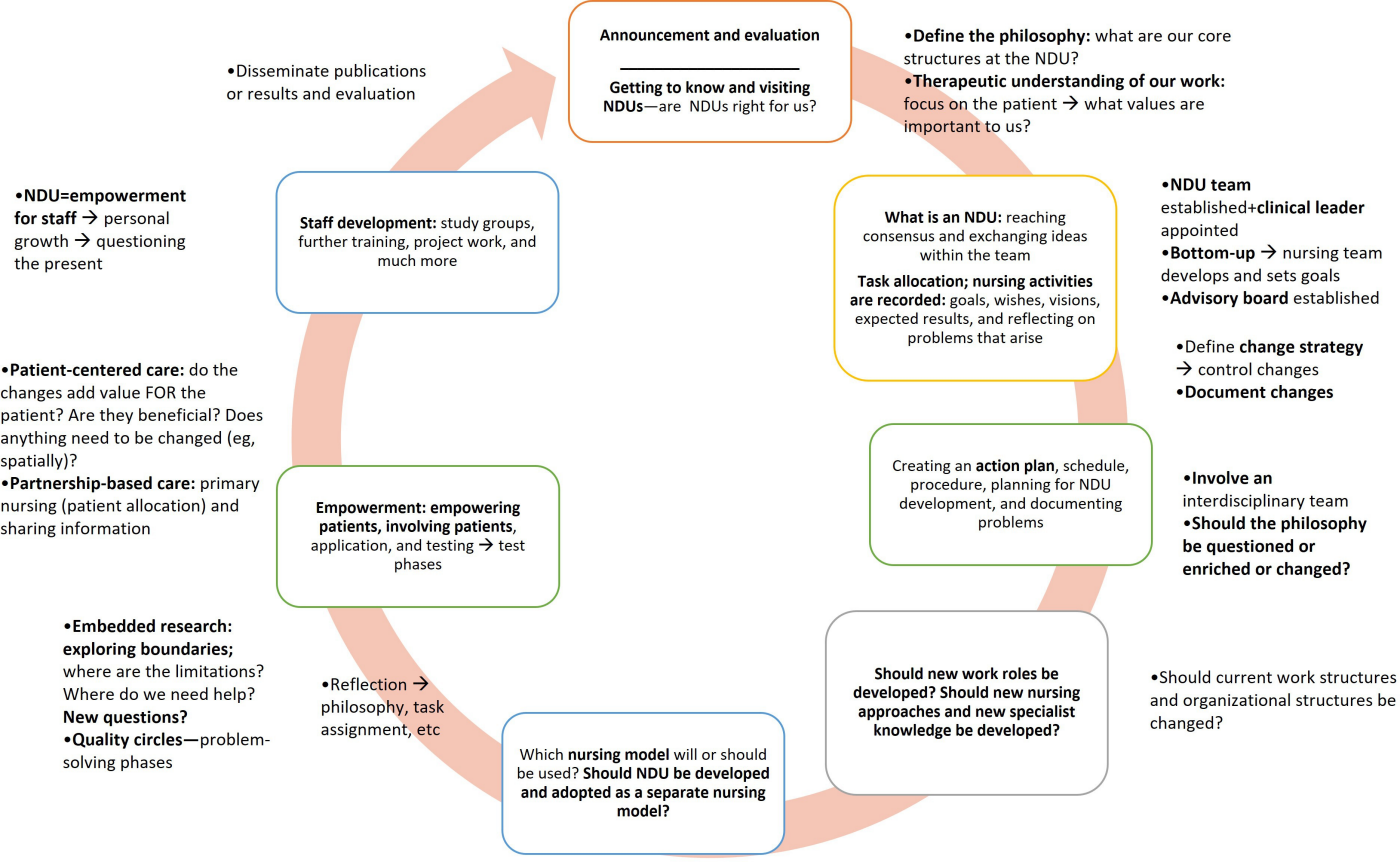
Progressive development toward a Nursing Development Unit (NDU) synthesized inductively from the included publications.

NDU development was reported as a multiyear, iterative process involving alignment of organizational structures, professional identity formation, and collaborative teamwork (Figure 4). Implementation evolves through cycles of reflection, strategic planning, practice-based experimentation, and evidence-informed evaluation grounded in a shared philosophy of practice that incorporates diverse staff perspectives and emphasizes holistic, patient-centered nursing [8,11,43,55].

Implementation practices reported included client-centered care, nurse empowerment, and evidence-based routines [11,29]. Adaptations to local cultural, staffing, and regional conditions were described as supporting flexibility while maintaining core principles [49]. Staff engagement in data collection and analysis was reported to facilitate learning, support innovation uptake, and foster interdisciplinary thinking [50,51,53].

Challenges reported in the literature included staff turnover, limited acceptance by other health care professionals, high workloads, and gaps between theory and practice. Sustained leadership support and team commitment were described as important factors for maintaining NDU development over time [28].

## Discussion

### Core Characteristics of NDUs: A Foundation for Innovation

This scoping review mapped the structural and procedural characteristics of NDUs across individual, interpersonal, and organizational levels based on 40 international publications from the United Kingdom and Australia spanning 1991 to 2017. The findings confirm NDUs as multilevel, practice-based innovation spaces that foster professional development, collaborative cultures, and structured learning. These characteristics are not merely descriptive but reflect a coherent framework for supporting continuous improvement and evidence-informed practice.

At the individual level, NDUs are not merely settings for skill development but spaces for professional identity formation. The emphasis on reflective practice, leadership identity, and resilience suggests that NDUs support nurses in navigating complex clinical realities and evolving roles [10,23,33,43]. This process fosters psychological safety and self-efficacy—conditions that are increasingly recognized as essential for adaptive learning and innovation [56,57].

At the interpersonal level, NDUs function as cocreation platforms. The documented practices of shared decision-making, collaborative planning, and peer learning indicate a culture where knowledge is coproduced rather than imposed in a top-down manner [23,32,45,50]. This participatory orientation aligns with contemporary models of human-centered design and may reduce resistance to change—especially when introducing new technologies [16,58].

At the organizational level, NDUs are defined by structured learning cycles and embedded evaluation systems. Their iterative development process—rooted in reflection, experimentation, and feedback—mirrors established frameworks for organizational change [10,11,29,50,51,53]. This suggests that NDUs are not static entities but dynamic systems capable of continuous adaptation, a quality that is critical in rapidly evolving digital health environments [59,60].

Crucially, these characteristics are not isolated features but interconnected processes that reinforce one another. For example, individual reflection is amplified through team dialogue, team collaboration is guided by organizational frameworks, and organizational learning is sustained through individual and team engagement. This synergistic interplay positions NDUs as potential organizational incubators for innovation—although the extent to which they support digital transformation remains to be empirically tested.

### Implications for Digital Transformation: A Cautious Interpretive Framework

While the included publications did not explicitly address digital technologies, the identified characteristics of NDUs suggest potential conditions that may support digital transformation in nursing. This section presents these connections as interpretive transfer hypotheses—plausible inferences based on conceptual alignment with known prerequisites for successful technology adoption.

NDUs may support digital transformation by fostering the professional and organizational capacities required for technology integration. For instance, reflective practice and continuous competence development—core to NDUs—can help nurses critically engage with digital tools, align them with ethical and clinical values, and integrate them meaningfully into care routines [33,34,43]. This supports the development of a digital professional identity where technology use is not seen as an external imposition but as an extension of nursing expertise [5,6,61,62].

The participatory culture of NDUs—characterized by shared decision-making, co-design, and peer learning—may reduce resistance to change and improve the fit of digital tools with clinical workflows [16,63-65]. By involving nurses in the design, testing, and evaluation of digital solutions, NDUs could serve as safe spaces for experimentation, where failures are reframed as learning opportunities [15,16]. This aligns with human-centered design principles and may enhance user acceptance and long-term sustainability [58].

At the organizational level, the structured frameworks for change management, iterative evaluation, and evidence-based practice in NDUs mirror the adaptive and learning-oriented processes needed for digital implementation [7,15,50,51]. The emphasis on feedback loops, strategic planning, and continuous improvement provides a robust infrastructure for managing the uncertainties and disruptions associated with digital innovation [60,66].

However, these implications must be interpreted with caution. The evidence base is limited in both temporal and technological scope: most included studies (39/40, 97.5%) were published before 2007, and none explicitly examined digital tools or competencies. Therefore, the claim that NDUs “support digital transformation” should not be understood as a proven causal relationship but rather as a plausible hypothesis that requires empirical validation in contemporary digital contexts.

### Strengths and Limitations

This review’s strength lies in its systematic and transparent approach guided by established frameworks (Joanna Briggs Institute and PRISMA-ScR guidelines). The inclusion of diverse study types and international literature provides a broad understanding of NDU development and implementation. The independent screening and iterative data extraction process enhances rigor, transparency, and generalizability.

Several limitations must be acknowledged. First, while this review focused on publications explicitly labeled as “Nursing Development Unit,” it is possible that related concepts or alternative labels (eg, “Clinical Development Unit” and “Practice Development Unit”) exist in more recent (digital) nursing literature and may share similar mechanisms for supporting innovation and technology adoption. Although supplementary searches were conducted, our decision to anchor the database search to the exact phrase “Nursing Development Unit” may have led to missed publications describing comparable models under different terminology. Consequently, our synthesis should be interpreted as mapping the literature that explicitly self-identified as “NDU” rather than exhaustively capturing all international Practice Development Unit models. Second, the age of the included publications (mostly published before 2007) limits the direct applicability of the findings to current digital health landscapes, where technologies such as artificial intelligence, real-time monitoring, and interoperable electronic health records are increasingly central. Third, the lack of outcome evaluations and the heterogeneity of definitions and implementation contexts reduce the comparability of the findings. Fourth, the exclusion of non–English- and non–German-language publications may have introduced language bias. Fifth, the absence of a formal quality appraisal—typical in scoping reviews that prioritize conceptual mapping over effect estimation—limits the comprehensive assessment of the robustness of individual findings.

These limitations underscore the need for future research to explicitly link NDU principles with digital transformation using contemporary case studies, mixed methods designs, and outcome-focused evaluations.

### Conclusions: A Foundation for Future Inquiry

This review demonstrates that NDUs are structured environments that promote professionalization, collaboration, and learning through multilevel processes. While the evidence base does not directly support claims about digital transformation, the core characteristics of NDUs—reflective practice, participatory cultures, and iterative learning—may offer enabling conditions for the adoption and integration of digital technologies in nursing.

However, these connections remain theoretical and require empirical testing. Future research should investigate how NDUs can be adapted to support digital innovation, with a focus on digital competencies, workflow integration, ethical considerations, and staff well-being. Only through such research can we determine whether NDUs are indeed a viable foundation for digital transformation in nursing or whether new models are needed to meet the demands of the digital age.

## Supplementary material

10.2196/89051 Multimedia Appendix 1 Search strategy.

10.2196/89051 Checklist 1 PRISMA-ScR checklist.

## Abbreviations

NDU: Nursing Development Unit
PRISMA: Preferred Reporting Items for Systematic Reviews and Meta-Analyses
PRISMA-ScR: Preferred Reporting Items for Systematic Reviews and Meta-Analyses extension for Scoping Reviews

## References

[1] Canfell OJ, Woods L, Meshkat Y (2024). The impact of digital hospitals on patient and clinician experience: systematic review and qualitative evidence synthesis. J Med Internet Res.

[2] Schlicht L, Wendsche J, Melzer M, Tschetsche L, Rösler U (2025). Digital technologies in nursing: an umbrella review. Int J Nurs Stud.

[3] Ruksakulpiwat S, Thorngthip S, Niyomyart A (2024). A systematic review of the application of artificial intelligence in nursing care: where are we, and what’s next?. J Multidiscip Healthc.

[4] Chua M, Lau XK, Ignacio J (2024). Facilitators and barriers to implementation of telemedicine in nursing homes: a qualitative systematic review and meta-aggregation. Worldviews Evid Based Nurs.

[5] Bimerew M (2024). Barriers and enablers of nurses’ adoption of digital health technology to facilitate healthcare delivery in resource-limited settings. Stud Health Technol Inform.

[6] Walzer S, Armbruster C, Mahler S, Farin-Glattacker E, Kunze C (2025). Factors influencing the implementation and adoption of digital nursing technologies: systematic umbrella review. J Med Internet Res.

[7] Gerrish K (2001). A pluralistic evaluation of nursing/practice development units. J Clin Nurs.

[8] Atsalos C, Greenwood J (2001). The lived experience of clinical development unit (nursing) leadership in Western Sydney, Australia. J Adv Nurs.

[9] Vaughan B (1998). The story of NDUs — how the nursing, midwifery and health visiting development unit programme began. NT Res.

[10] Bland A (1997). Developing the emergency nurse practitioner role in accident and emergency: a bottom-up approach. Accid Emerg Nurs.

[11] Parsons M, Mott S (2003). Royal Rehabilitation Centre Sydney: towards Clinical Development Units (Nursing). Collegian.

[12] Greenwood J (1997). Should nursing development units be accredited?. Collegian.

[13] Greenwood J (2000). Clinical development units (nursing): Issues surrounding their establishment and survival. Int J Nurs Pract.

[14] Graham ID, Logan J, Harrison MB (2006). Lost in knowledge translation: time for a map?. J Contin Educ Health Prof.

[15] Pomare C, Churruca K, Long JC, Ellis LA, Braithwaite J (2019). Organisational change in hospitals: a qualitative case-study of staff perspectives. BMC Health Serv Res.

[16] Lyon AR, Munson SA, Renn BN (2019). Use of human-centered design to improve implementation of evidence-based psychotherapies in low-resource communities: protocol for studies applying a framework to assess usability. JMIR Res Protoc.

[17] von Elm E, Schreiber G, Haupt CC (2019). Methodische anleitung für scoping reviews (JBI-methodologie) [Article in German]. Z Evid Fortbild Qual Gesundhwes.

[18] Arksey H, O’Malley L (2005). Scoping studies: towards a methodological framework. Int J Soc Res Methodol.

[19] Tricco AC, Lillie E, Zarin W (2018). PRISMA extension for scoping reviews (PRISMA-ScR): checklist and explanation. Ann Intern Med.

[20] Kuckartz U, Rädiker S (2024). Qualitative Inhaltsanalyse. Methoden, Praxis, Umsetzung mit Software und Künstlicher Intelligenz.

[21] Levac D, Colquhoun H, O’Brien KK (2010). Scoping studies: advancing the methodology. Implement Sci.

[22] Moher D, Liberati A, Tetzlaff J, Altman DG, PRISMA Group (2009). Preferred reporting items for systematic reviews and meta-analyses: the PRISMA statement. PLoS Med.

[23] Atsalos C, O’Brien L, Jackson D (2007). Against the odds: experiences of nurse leaders in Clinical Development Units (Nursing) in Australia. J Adv Nurs.

[24] Avallone I, Gibbon B (1998). Nurses’ perceptions of their work environment in a Nursing Development Unit. J Adv Nurs.

[25] Bell M, Procter S (1998). Developing nurse practitioners to develop practice: the experiences of nurses working on a nursing development unit. J Nurs Manag.

[26] Bowles A, Bowles NB (2000). A comparative study of transformational leadership in nursing development units and conventional clinical settings. J Nurs Manag.

[27] Cannard G (1996). The effect of aromatherapy in promoting relaxation and stress reduction in a general hospital. Complement Ther Nurs Midwifery.

[28] Christensen M, Craft J (2017). The nursing professorial unit: translating acute and critical care nursing research. Int Pract Dev J.

[29] Christian SL, Norman IJ (1998). Clinical leadership in nursing development units. J Adv Nurs.

[30] Draper J (1996). Nursing development units: an opportunity for evaluation. J Adv Nurs.

[31] Duffield C (2005). A Master Class for nursing unit managers: an Australian example. J Nurs Manag.

[32] Gerrish K, Ferguson A (2000). Nursing development units: factors influencing their progress. Br J Nurs.

[33] Graham I (1996). A presentation of a conceptual framework and its use in the definition of nursing development within a number of nursing development units. J Adv Nurs.

[34] Graham IW (2000). Reflective practice and its role in mental health nurses’ practice development: a year-long study. J Psychiatr Ment Health Nurs.

[35] Graham I (2003). Leading the development of nursing within a Nursing Development Unit: the perspectives of leadership by the team leader and a professor of nursing. Int J Nurs Pract.

[36] Greenwood BJ (2000). On donkeys, ingratiates, thinkers and...clinical development units. Collegian.

[37] Greenwood J (1999). Clinical Development Units (Nursing): the Western Sydney approach. J Adv Nurs.

[38] Greenwood J, Kearns E (1996). Establishing a transcultural nursing development unit: the South-Western Sydney experience. Collegian.

[39] Greenwood J, Parsons M (2002). The evaluation of a clinical development unit leadership preparation program by focus group interviews - part 2: negative aspects. Nurse Educ Today.

[40] Happell B, Martin T (2002). Changing the mental health nursing culture: the nursing clinical development unit approach. Int J Ment Health Nurs.

[41] Happell B, Martin T (2004). Exploring the impact of the implementation of a nursing clinical development unit program: what outcomes are evident?. Int J Ment Health Nurs.

[42] Happell B, Martin T (2005). Changing the culture of mental health nursing: the contribution of nursing clinical development units. Issues Ment Health Nurs.

[43] Johns C (1991). The Burford Nursing Development Unit holistic model of nursing practice. J Adv Nurs.

[44] Keatinge D, Scarfe C (1998). Creating a nursing development unit in a dementia care context. Int J Nurs Pract.

[45] Keatinge D, Scarfe C, Bellchambers H (2000). The manifestation and nursing management of agitation in institutionalised residents with dementia. Int J Nurs Pract.

[46] Malby R (1996). Nursing development units in the United Kingdom. Adv Pract Nurs Q.

[47] Manley K (1997). A conceptual framework for advanced practice: an action research project operationalizing an advanced practitioner/consultant nurse role. J Clin Nurs.

[48] Pearson A (1997). An evaluation of the King’s Fund Centre Nursing Development Unit network 1989-91. J Clin Nurs.

[49] Redfern S, Stevens W (1998). Nursing development units: their structure and orientation. J Clin Nurs.

[50] Redfern S, Murrells T (1998). Research, audit and networking activity in nursing development units. NT Research.

[51] Redfern S, Normand C, Christian S (1997). An evaluation of nursing development units. NT Res.

[52] Ryan A (1994). Improving discharge planning. Nurs Times.

[53] Schiereck S (2000). Social interaction between nurses and female patients in the course of organization of a Nursing Development Unit [Article in German]. Pflege.

[54] Scholes J (1996). Role transition and emotional labour: understanding the impact of a nursing development unit on staff and on the therapeutic milieu. NT Res.

[55] Wright SG (2007). Developing nursing: the contribution to quality. 1988. Int J Health Care Qual Assur.

[56] Bektaş G, Ünal BÖ, Nal M (2025). The effect of transformational leadership on nurses’ innovative behaviors: the mediating effect of psychological empowerment. BMC Nurs.

[57] Elbus LM, Mostafa MG, Mahmoud FZ, Shaban M, Mahmoud SA (2024). Nurse managers’ managerial innovation and it’s relation to proactivity behavior and locus of control among intensive care nurses. BMC Nurs.

[58] Dopp AR, Parisi KE, Munson SA, Lyon AR (2019). Integrating implementation and user-centred design strategies to enhance the impact of health services: protocol from a concept mapping study. Health Res Policy Syst.

[59] Øygarden O, Olsen E, Mikkelsen A (2020). Changing to improve? Organizational change and change-oriented leadership in hospitals. J Health Organ Manag.

[60] van den Hoed MW, Daniëls R, Beaulen A, Hamers JP, van Exel J, Backhaus R (2024). Perspectives on managing innovation readiness in long-term care: a Q-methodology study. BMC Geriatr.

[61] De Leeuw JA, Woltjer H, Kool RB (2020). Identification of factors influencing the adoption of health information technology by nurses who are digitally lagging: in-depth interview study. J Med Internet Res.

[62] Ramadan OM, Elsharkawy NB, Hafiz AH (2025). Neonatal nurses’ e-health literacy and technology‑mediated clinical practice: a cross-sectional analysis of digital health competencies and practice patterns. BMC Nurs.

[63] Kyrarini M, Lygerakis F, Rajavenkatanarayanan A (2021). A survey of robots in healthcare. Technologies.

[64] Silvera-Tawil D (2024). Robotics in healthcare: a survey. SN Comput Sci.

[65] Trainum K, Tunis R, Xie B, Hauser E (2023). Robots in assisted living facilities: scoping review. JMIR Aging.

[66] Ignatowicz A, Tarrant C, Mannion R, El-Sawy D, Conroy S, Lasserson D (2023). Organizational resilience in healthcare: a review and descriptive narrative synthesis of approaches to resilience measurement and assessment in empirical studies. BMC Health Serv Res.

