# Supplementary material for: Facilitating Digital Transformation in Nursing Through Nursing Development Units: Scoping Review
Source: JMIR Nurs. 2026 Jun 4;9:e89051. doi: 10.2196/89051 (PMC13235844; doi:10.2196/89051)
Supplement: Multimedia Appendix 1 [file nursing-v9-e89051-s001.docx]

**Multimedia Appendix 1. Search Strategy**

| Database: | MEDLINE (via PubMed) |
| --- | --- |
| Search date: | May 2025 |
| Search strategy: | "nursing development unit" |
| Filters applied: | none |
| Time restrictions: | none |
| Language restrictions: | none |

*The same search strategy was applied across all databases.*
